# Pyruvate oxidase of *Streptococcus pneumoniae* contributes to pneumolysin release

**DOI:** 10.1186/s12866-016-0881-6

**Published:** 2016-11-09

**Authors:** Joseph C. Bryant, Ridge C. Dabbs, Katie L. Oswalt, Lindsey R. Brown, Jason W. Rosch, Keun S. Seo, Janet R. Donaldson, Larry S. McDaniel, Justin A. Thornton

**Affiliations:** 1 Department of Biological Sciences, Mississippi State University, 295 E Lee Blvd., Harned Hall, Rm 219, Mississippi State, MS 39762 USA; 2 Department of Infectious Diseases, St. Jude Children’s Research Hospital, Memphis, TN USA; 3 Department of Basic Sciences, College of Veterinary Medicine, Mississippi State University, Mississippi State, MS USA; 4 Department of Biological Sciences, University of Southern Mississippi, Hattiesburg, MS USA; 5 Department of Microbiology and Immunology, University of Mississippi Medical Center, Jackson, MS USA

**Keywords:** *Streptococcus pneumoniae*, Pneumococcus, Pneumolysin, Virulence, Toxin, Metabolism, Protein secretion, Cytotoxicity

## Abstract

**Background:**

*Streptococcus pneumoniae* is one of the leading causes of community acquired pneumonia and acute otitis media. Certain aspects of *S. pneumoniae*’s virulence are dependent upon expression and release of the protein toxin pneumolysin (PLY) and upon the activity of the peroxide-producing enzyme, pyruvate oxidase (SpxB). We investigated the possible synergy of these two proteins and identified that release of PLY is enhanced by expression of SpxB prior to stationary phase growth.

**Results:**

Mutants lacking the *spxB* gene were defective in PLY release and complementation of *spxB* restored PLY release. This was demonstrated by cytotoxic effects of sterile filtered supernatants upon epithelial cells and red blood cells. Additionally, peroxide production appeared to contribute to the mechanism of PLY release since a significant correlation was found between peroxide production and PLY release among a panel of clinical isolates. Exogenous addition of H_2_O_2_ failed to induce PLY release and catalase supplementation prevented PLY release in some strains, indicating peroxide may exert its effect intracellularly or in a strain-dependent manner. SpxB expression did not trigger bacterial cell death or LytA-dependent autolysis, but did predispose cells to deoxycholate lysis.

**Conclusions:**

Here we demonstrate a novel link between *spxB* expression and PLY release. These findings link liberation of PLY toxin to oxygen availability and pneumococcal metabolism.

**Electronic supplementary material:**

The online version of this article (doi:10.1186/s12866-016-0881-6) contains supplementary material, which is available to authorized users.

## Background

*S. pneumoniae* (pneumococcus) is a Gram-positive human pathogen identified as a cause of acute otitis media, bacteremia, septicemia, pneumonia, and meningitis [1] and is the leading cause of death in children under the age of five worldwide [2]. Diseases resulting from pneumococcal infection impart a major economic impact, with healthcare costs estimated to range from 3 billion to 6 billion dollars annually in the United States for otitis alone [3]. In spite of this major disease burden, it most often exists as a commensal organism of the nasopharynx, with carriage rates of up to 70 % depending on the demographic [4]. Pneumococcus produces and secretes a number of surface proteins which contribute to virulence including neuraminidase, hyaluronidase, and pneumococcal surface protein A (PspA) [5–7]. However, it produces relatively few protein exotoxins compared to other pathogenic species capable of such invasive disease.

The primary toxin expressed by *S. pneumoniae* is pneumolysin (PLY), which is a 53-kDa cholesterol dependent pore-forming cytolysin (CDC) [8]. PLY has been found to reduce ciliary beating within the lungs and deletion of the *ply* gene from the pneumococcal chromosome attenuates virulence in vivo [9]. Additionally, exposure to PLY activates differential gene expression within host cells [10]. Unlike the other members of the CDC family, PLY lacks an N-terminal signal sequence for extracellular release via the Sec-dependent pathway [11]. Despite this, it is well established that PLY is released into the extracellular space [8, 9, 12].

Controversy exists as to the primary route PLY takes to exit the cytoplasm. The mechanism of release was long thought to be solely attributable to autolysis of the bacterial cell [13]. However, PLY release has been demonstrated in the absence of autolysis [14], suggesting that other mechanisms must contribute to liberation of PLY during pneumococcal growth. Other findings have demonstrated that PLY can actually traverse and associate with the pneumococcal cell wall [15]. Subsequent studies by Price et al. demonstrated that domain 2 of PLY is essential for the cell wall association and that the export pathway was conserved in *Bacillus subtilis* [12]. The composition of the pneumococcal peptidoglycan has been recently shown to restrict PLY release [16]. Interestingly, this study found that greater PLY release does not correlate with enhanced virulence and that rather a controlled release of PLY is important for pathogenesis. These findings underline the fact that, while capable of inflicting damage to the host, PLY is also a stimulator of host immune responses [17].

*S. pneumoniae* is unique among catalase-negative organisms due to the fact that it produces up to millimolar concentrations of hydrogen peroxide (H_2_O_2_), primarily through the activity of the enzyme pyruvate oxidase (SpxB) [18, 19]. In aerobic environments, pneumococcus utilizes SpxB to convert pyruvate to acetate, a reaction that produces acetyl phosphate, CO_2_, and H_2_O_2_ [20]. Although SpxB-derived H_2_O_2_ is detrimental to survival of the pneumococcus at high concentrations, deletion of *spxB* has been shown to reduce virulence in vivo [21]. Pneumococcal H_2_O_2_ has also been shown to aid the pneumococcus in competing with other inhabitants of the upper respiratory tract [22] and possibly has a significant impact upon host cells and tissues. It is cytotoxic to numerous cell types including neuronal cells, neutrophils, and alveolar epithelial cells [23–26]. During stationary phase, pneumococcal H_2_O_2_ results in pneumococcal cell death resembling apoptosis of eukaryotic cells and this process does not require the major autolysin, *N*-acetylmuramoyl-l-alanine amidase (LytA) [27]. However, strains vary in their production of H_2_O_2_, with some strains producing significant concentrations prior to stationary phase. The impact of low and intermediate levels of H_2_O_2_ upon the physiology and structural integrity of *S. pneumoniae* has not been investigated in detail. Price et al. demonstrated PLY associating with the cell wall [15], indicating that by some mechanism PLY escapes the cell membrane. Additionally, pneumococcus is known to alter its membrane composition in response to endogenous reactive oxygen species [28] Based on these results, we hypothesized that pneumococcal H_2_O_2_ might have non-lethal effects on the physiology of the bacterium at early phases of growth, possibly affecting release of PLY. We investigated the impact of SpxB on PLY localization into bacterial supernatants and the effect of this upon host cell integrity.

## Methods

### Bacterial strains and culture conditions

*S. pneumoniae* strains TIGR4 [29], an unencapsulated mutant of TIGR4 (T4R) [30], WU2 [31], AW267, along with isogenic mutants of these strains were grown in Todd Hewitt media plus 0.5 % yeast extract (THY) to a mid-logarithmic phase of growth (OD_600_ of 0.5) at 37 °C. Clinical isolates were received from the Center for Disease Control and Prevention’s Active Bacterial Core surveillance (ABCs) isolate bank (http://www.cdc.gov/abcs/index.html). For additional studies strains lacking the major cell wall amidase LytA (Δ*lytA*) were created as described below. The ∆*lytA* isogenic mutants of T4R and WU2, along with ∆*lytA∆spxB* double mutants were cultured under the same conditions as parental strains. To neutralize pneumococcal H_2_O_2_ from supernatants, THY media was supplemented with catalase derived from *Aspergillus niger* (2 ng/mL; cat #C3515 Sigma Aldrich). A549 type 2 lung epithelial cells (ATCC) were cultured in F12-K medium (ATCC) supplemented with 10 % fetal bovine serum (FBS) at 37 °C in 5 % CO_2_ atmosphere.

### Mutant construction

Isogenic ∆*spxB* mutants were developed in the strains TIGR4, WU2, T4R, and AW267 by allelic replacement. Briefly, the *spxB* gene with 500 base pairs flanking each end was amplified from T4R chromosomal DNA using primers ALR1 and ALR2, designed to contain both KpnI and XbaI restriction sites. The product was digested with KpnI and XbaI restriction endonucleases (New England BioLabs, Ipswich, MA) and ligated into KpnI and XbaI digested pBluescript vector using T4 DNA ligase (Thermo Fisher Scientific). Inverse PCR was performed using primers ALR3 and ALR4 to amplify outward from just inside the coding sequence of *spxB* from a positive clone. Primers ALR3 and ALR4 were designed to include BamHI sites. The gene *ermB* was amplified using designed primers ALR5 and ALR6 including BamHI sites and both products were digested with BamHI (New England BioLabs, Ipswich, MA) overnight at 37 °C. The linearized vector that was amplified with primers ALR3 and ALR4 and the *ermB* insert amplified with primers ALR5 and ALR6 were ligated and transformed into DH5α *E. coli* cells. Positive colonies with bands of the appropriate size were grown in LB medium overnight and frozen at −80 °C. PCR products containing the knockout construct were amplified from positive clones and used to transform pneumococcal strains by standard methods and subsequent selection for transformants by plating on blood agar plates containing erythromycin (0.5 μg/mL). Mutants lacking the *lytA* gene were generated in strains T4R and WU2 by overlap extension PCR mutagenesis as previously described [32]. Briefly, 500–1000 base pair DNA sequences flanking each side of the *lytA* gene were amplified using primers LytA-KO1, LytASup LytASdn, and LytA-KO4 (Table 1) and fused by PCR to the spectinomycin resistance cassette amplified from the shuttle vector pNE-1. The *lytA* gene was replaced with a spectinomycin cassette following transformation of *S. pneumoniae* strains by standard methods and selected for by plating on blood agar plates supplemented with spectinomycin (500 μg/mL). The T4R ∆*ply* mutant was created using the same method (with primers PlyAF, PlyAR, PlyBF, PlyBR), but replacing the *ply* gene with an erythromycin cassette. Primer sequences for all mutants are listed in Table 1. Complemented mutants in strains TIGR4 and T4R were developed through cloning the *spxB* gene from T4R by amplifying the gene by PCR with primers SpxB-F and SpxB-R followed by digestion of the product with EcoRI and XmaI enzymes (New England Biolabs, Ipswich, MA). Following digestion of the product, the gene was ligated with the pNE-1 pneumococcal shuttle vector that was similarly digested. The ligation was transformed into *E. coli* strain DH5α. Purified plasmid was then used to transform ∆*spxB* strains by standard methods and complemented mutants were selected by plating on blood agar plates supplemented with erythromycin (0.5 μg/mL) and spectinomycin (500 μg/mL). Complementation of *spxB* was confirmed by PCR and by assaying supernatant from clones by a Pierce Quantitative Peroxide Assay (cat# 23280; Life Technologies).

**Table 1:** Primer sequences

Primer name	Sequence (5′-3′)
LytA-KO1	GCGGGTACCCAGTCCAGCTTTGGTTTCCT
LytASup	TAAAAATATCTCTTGCCAGTCCTTGCCTATATGGTTGCACG
LytASdn	GGTAATCAGATTTTAGAAAACAATAAACCCTCACAGTAGAGCCAGAT
LytA-KO4	CGCGGATCCTCACAGTAGAGCCAGATGGC
PlyAF	CTCAATCCAGCTACCTGTCGC
PlyAR	GTTTGCTTCTAAGTCTTATTTCCCTTCTACCTCCTAATAAG
PlyBF	GAGTCGCTTTTGTAAATTTGGGAGAGGAGAATGCTTGCG
PlyBR	GCTTGTTTAGCACGGTCG
ALR1	GCG GGTACCGCGTGCTATTGCAGATCAAA
ALR2	GCGTCTAGACATCGTTAATCGGAGATGGA
ALR3	CGCGGATCCATCTACGCCCCATGTTTTCAATACG
ALR4	CGCGGATCCACCATTCCGTCTCTTCTTGG
ALR5	CGCGGATCCGGAAATAAGACTTAGAAGCAAAC
ALR6	CGCGGATCCCCAAATTTACAAAAGCGACTC
SpxB-F	CGCGCCCGGGTGACAACACTTTCAAAACTG
SpxB-R	CGCGGAATTCTTATTTAATTGCGCGTGATTGC
ERM-F	GGAAATAAGACTTAGAAGCAAAC
ERM-R	CCAAATTTACAAAAGCGACTC
Spec-F	CGTGACTGGCAAGAGATATTTTTA
Spec-R	GGGTTTATTGTTTTCTAAAATCTGATTACC
PLY-F	CAGAGCGTCCTTTGGTCTATATT
PLY-R	CAGCCTCTACTTCATCACTCTTAC

### H_2_O_2_ quantitation

Pneumococci were grown to mid-log phase (OD_600_ of 0.5). Following centrifugation (16,000 × g for 5 min) of 1 mL of the culture, the supernatant was subsequently filtered through a 0.22 μm polyethersulfone (PES) membrane syringe filter (CellTreat) to remove remaining bacterial cells and analyzed for hydrogen peroxide production using a colorimetric hydrogen peroxide quantification assay per manufacturer instructions in a BioTek Synergy HT plate reader at an OD_540_ (cat# 23280; Life Technologies). This analysis was performed in triplicate.

### H_2_O_2_ Treatment

To ascertain the impact of H_2_O_2_ alone on the release of PLY, T4R and its isogenic Δ*spxB* mutant were grown to mid log phase (OD_600_ of 0.5) in 5 mL of THY media and treated with either 0 μM or 500 μM H_2_O_2_. Samples were then placed on ice for 1 h, after which H_2_O_2_ was quantified following centrifugation of 1 mL of culture at 15,000 rpm for 5 min and filtration of the supernatant through PES (CellTreat) syringe filters as described above. Bacterial counts were determined before and after the H_2_O_2_ treatment by viable plate counts using blood agar plates.

### Western blot

To determine the relative amount of PLY released between parental and mutant strains, sterile-filtered bacterial supernatants from cultures grown to mid-log phase (OD_600_ of 0.5) were denatured by boiling for 5 min and separated on 10 % SDS-PAGE gels (Bio-Rad) prior to being transferred onto a 0.22 nm PVDF membrane (Millipore). The membranes were then blocked for 30 min (5 % milk) and probed with rabbit polyclonal anti-PLY antibody overnight. The blot was subsequently washed with and probed with goat anti-rabbit HRP conjugated secondary antibody (BioRad) for 1 h. Blots were incubated with Luminata Forte substrate (Millipore) for 1 min at 25 °C. The membranes were then developed following exposure to radiography film (GeneMate). Band density was calculated using ImageJ software after scanning film at 600 dpi (NCBI).

### Dot blot

To quantitate PLY in the supernatants, recombinant PLY was serially diluted into THY media to create a standard of known concentration (20 ng to 5 μg). PLY standards (20 μL) and bacterial supernatants (100 μL) were applied onto PVDF membranes using a dot blotting vacuum manifold (Bio-Rad). Following 45 min of gravity filtration, light vacuum was applied to adhere the protein to the membrane and the wells were washed twice using 200 μL of phosphate buffered saline (PBS). The membrane was then blocked for 30 min in 5 % milk. The membrane was then probed with an anti-PLY rabbit polyclonal antibody at a 1:200 dilution overnight. Following exposure to the primary antibody, the blot was then probed with a goat anti-rabbit secondary for 1 h. The blot was developed using Luminata Forte substrate (Millipore) and exposed to X-ray film (GeneMate). Dot density was quantitated using ImageJ (NCBI) and then plotted against the known concentration of standards to determine quantity of PLY per 100 μL.

### Real time PCR

Pneumolysin gene expression in T4R, WU2, AW267, and their respective isogenic ∆*spxB* mutants was quantitated by qRT-PCR. Bacterial strains were grown to an OD_600_ of 0.5 in THY. Bacterial RNA was extracted following sonication of the bacterial culture, and a total of 4 min of bead beating with 0.1 mm zircon beads. Bacterial RNA was purified using the Qiagen RNeasy kit, with the inclusion of an on column RNase-free DNase treatment for 1 h. Bacterial RNA was quantitated using a Qubit fluorometer and 50 ng was used to generate cDNA utilizing a Maxima cDNA synthesis kit for qRT-PCR (Life Technologies). Gene-specific primer sequences PLY-F and PLY-R are listed in Table 1. The fold-change in gene expression was calculated using the ∆∆C_T_ method utilizing *gyrA* as an internal control.

### Flow cytometry

To assess the effect of bacterial supernatants upon human cells, 1 × 10^5^ A549 epithelial cells were treated with filtered bacterial supernatant obtained from T4R, T4R∆*spxB*, T4R∆*ply*, and the complemented T4R∆*spxB* (∆*spxB*+). Briefly, epithelial cells were pelleted by centrifugation at 380xg for 4 min, suspended in 0.2 mL of filtered bacterial supernatant, and incubated at 37 °C for 30 min on a rotating platform. Cells were then pelleted at 380 × g for 4 min and suspended in 0.5 mL PBS with 3 μg/mL propidium iodide (PI) (Sigma Aldrich). The cells were assessed using an Attune Flow Cytometer (Life Technologies) and the fluorescence intensity shift in the BL3 channel was measured as an indicator of epithelial cell death.

### Hemolysis assay

PLY release was quantitated by standard hemolytic assay. Briefly, bacterial supernatants from cultures grown to an OD_600_ of 0.3 were serially diluted (1:3) across the microtiter plate into phosphate-buffered saline (PBS) containing 0.1 % dithiothreitol (Sigma, St. Louis, MO). Washed 1 % sheep red blood cells in PBS were added to the wells and incubated at 37 °C for 30 min. After incubation, the sheep erythrocytes were pelleted and plates were imaged.

### Extracellular DNA quantitation

Bacterial strains were grown in THY medium to an OD_600_ of 0.5 and extracellular DNA was quantitated from sterile filtered supernatant by a Qubit fluorometer using Qubit dsDNA HS Assay Kit (Thermo Fisher Scientific).

### Autolysis

*S. pneumoniae* T4R and the isogenic ∆*spxB* mutant were grown in 12 mL of THY at 37 **°**C. Upon reaching OD_600_ 0.45, each 12 mL culture was split into three separate tubes. The separate tubes containing mid-log phase bacteria were incubated at room temperature until reaching an OD_600_ of 0.5. Upon reaching the desired OD, Triton X-100 (0.5 %) or sodium deoxycholate (0.05 %) was added to the respective culture tubes and the OD_600_ of the cultures was recorded every minute for a total of 10 min for each of the three culture tubes. Controls received no detergents. Additional control and experimental samples were grown in the presence of catalase (2 μg/mL).

Statistical analysis. All statistical analyses (Student’s *t*-test (two-tailed) and linear best-fit regression) were performed using GraphPad Prism software (www.graphpad.com) with a *p*-value < 0.5 considered significant.

## Results

### Expression of SpxB enhances PLY-dependent cell death

**Fig. 1:**
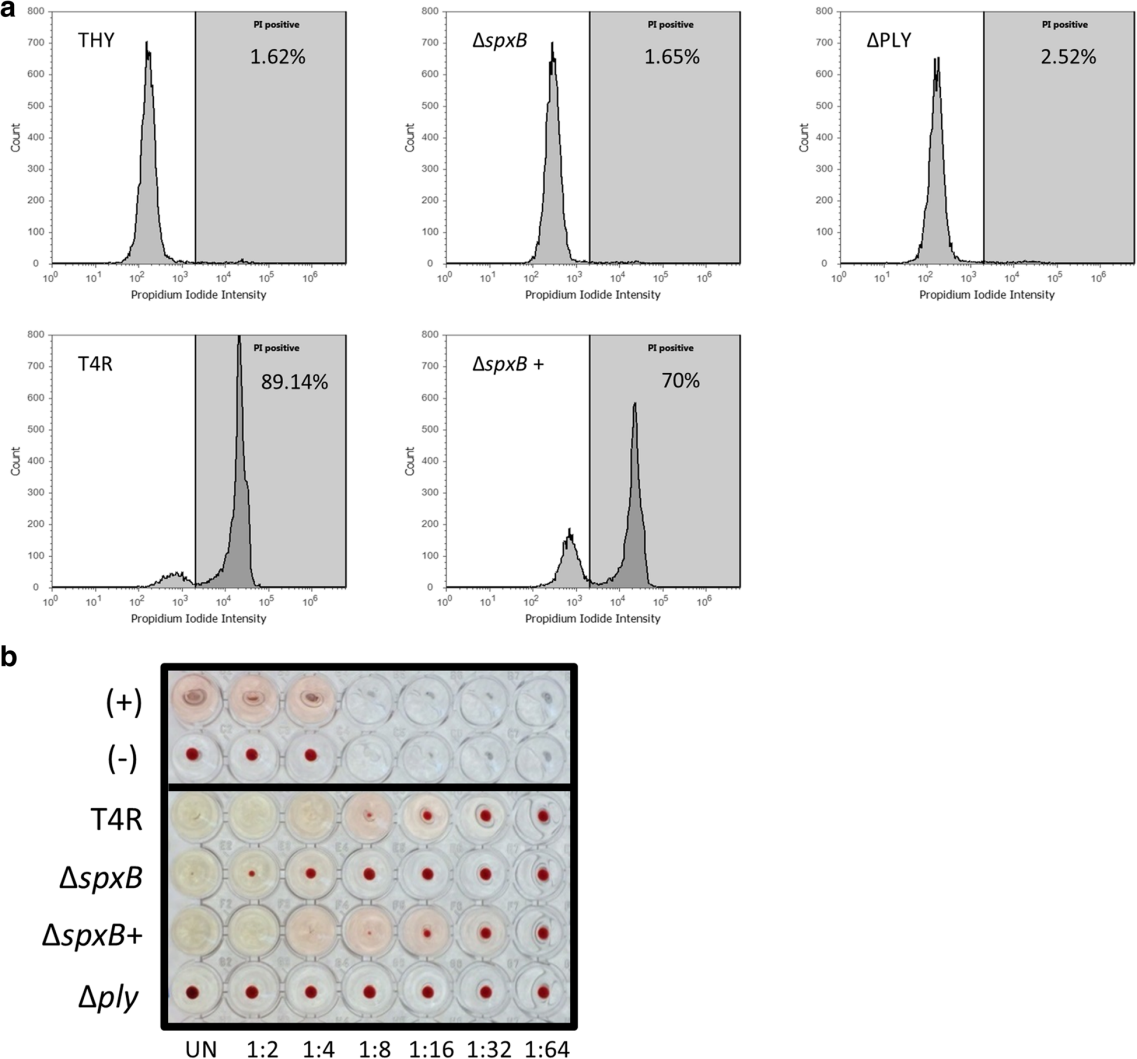
Loss of SpxB reduces cytotoxtic potential of culture supernatants. **a** A549 human epithelial cells were exposed to culture supernatants from parental strain T4R and mutant strains lacking SpxB or PLY. Cytotoxicity was assesed by measuring propidium iodide uptake using flow cytometry. **b** Hemolytic effect of PLY released in culture supernatant. Serially diluted culture supernatants from T4R and mutant strains lacking SpxB were incubated with washed sheep red blood cells for 30 min and subsequently centrifuged. The absence of a red pellet at the bottom of well indicates lysis of RBCs. (+) indicates distilled water positive control for complete hemolysis and (−) received no PLY. Results are representative of duplicate experiments

To determine the relative contribution of H_2_O_2_ and PLY secreted by pneumococcus to cytolysis of airway epithelial cells, A549 human lung epithelial cells were exposed to filter-sterilized supernatants collected from mid-log phase cultures of *S. pneumoniae* and isogenic mutants lacking either *spxB* or *ply* genes and the loss of membrane integrity was assessed by uptake of PI. Compared to THY media alone, exposure to supernatant from the parental strain T4R led to a nearly complete loss of A549 cell viability (89 %), while exposure to supernatant from a strain lacking pyruvate oxidase (T4R∆*spxB*) resulted in minimal loss in membrane integrity (1.65 %; Fig. 1a). This effect was shown to be dependent upon release of PLY into the supernatant since supernatant from a strain containing a functional *spxB* gene but lacking the *ply* gene (T4R∆*ply*) resulted in only 2.52 % of cells staining PI positive. Complementation of *spxB* (TR4∆*spxB*+) restored the percentage of dead cells to nearly that of A549 cells treated with T4R supernatant (70 % vs. 89 %, respectively). Similar results were obtained using a standard hemolysis assay, with supernatant from strains expressing PLY and SpxB leading to greater hemolysis than T4R∆*spxB* (Fig. 1b) Strains lacking both SpxB and PLY had no hemolysis, comparable to T4R∆*ply* (data not shown). These data demonstrate that expression of SpxB contributes to PLY-dependent cytotoxicity at mid-log phase growth.

### SpxB is responsible for the production of H_2_O_2_ among *S. pneumoniae* isolates at mid-log phase

*S. pneumoniae* is known to produce up to millimolar concentrations of H_2_O_2_ during growth [18]. The enzyme pyruvate oxidase (SpxB) is known to be responsible for the majority of peroxide production by pneumococcus grown aerobically [21]. However, the enzyme lactate oxidase is also capable of producing H_2_O_2_ [33]. We assessed the relative production of H_2_O_2_ among a panel of laboratory strains. Pneumococcal strains T4, T4R, WU2, and AW267 and their isogenic ∆*spxB* mutants were grown to mid-log phase (OD_600_ of 0.5) in THY and H_2_O_2_ was quantitated from filtered supernatants (Additional file 1: Figure S1 A-D). Peroxide concentrations were found to vary between strains. However, the deletion of *spxB* in all strains examined resulted in a significant reduction of H_2_O_2_ (*P* < 0.005), which was comparable to supernatants from cultures grown in the presence of catalase. These results demonstrated that SpxB is primarily responsible for the production of the H_2_O_2_ by the strains examined in this study during mid-log phase growth.

### SpxB enhances PLY release among *S. pneumoniae* isolates

**Fig. 2:**
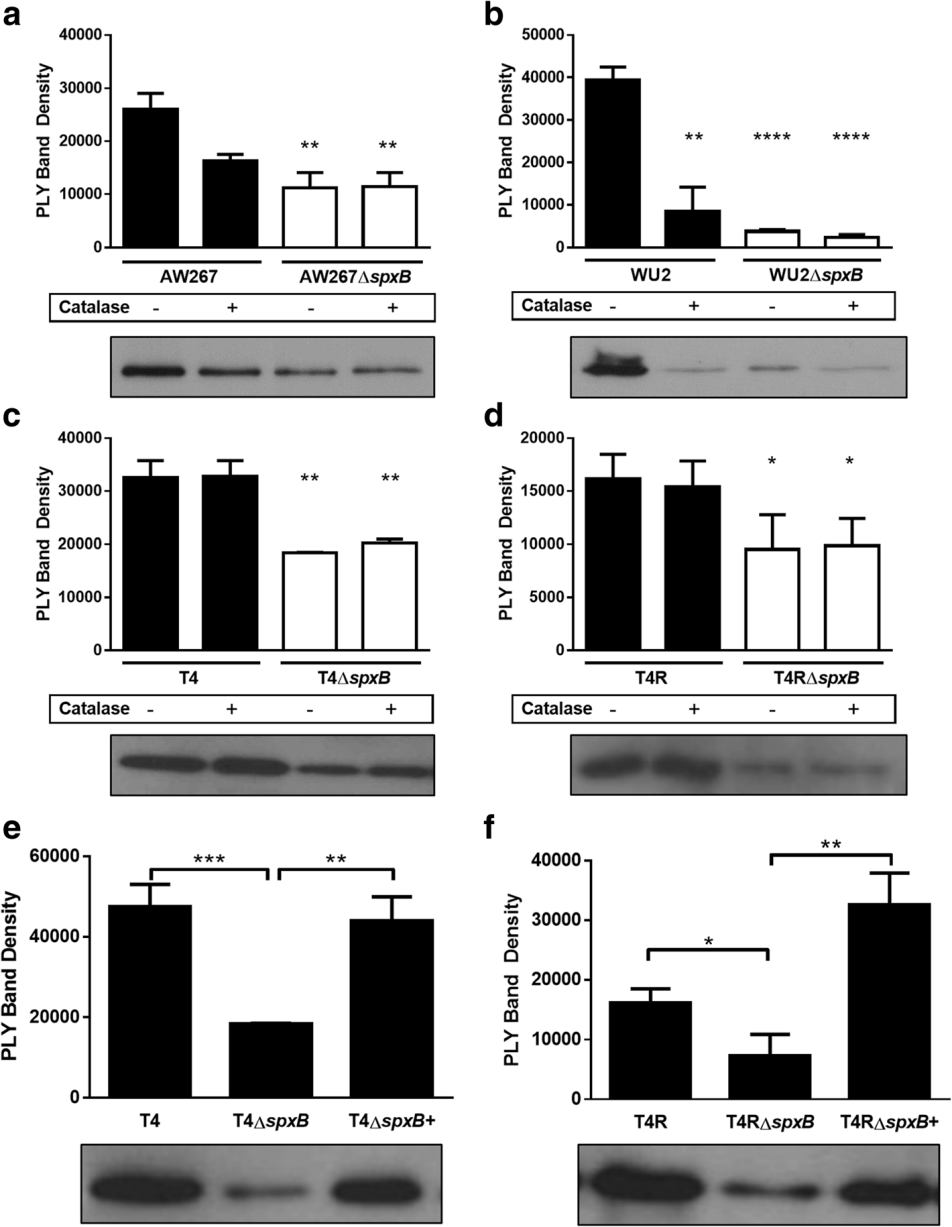
Deletion of *spxB* greatly reduces PLY observed in the supernatant. **a-d** The amount of PLY released into culture supernatants from parental and mutant strains lacking SpxB (**a** AW267; **b** WU2, **c** T4, and **d** T4R) was measured by western blot. Band intensities were quantitated by densitometry. Results are representative of three independent experiments ± SD. (* *p* < 0.05, ** *p* < 0.005, **** *P* < 0.00005). Where indicated, the cultures were complemented with 10 ng of catalase. **e** and **f** The amount of PLY released in culture supernatants from parental, mutant strains lacking SpxB, and complemented strains (**e** T4; **f** T4R) was measured by western blot. Band intensities were quantitated by densitometry. Results are representative of three independent experiments ± SD. (* *p* < 0.05, ** *p* < 0.005, *** *P* < 0.0005). Representative images of western blots are shown

To determine the contribution of SpxB upon PLY release, supernatants from TIGR4, T4R, WU2, and AW267, and their isogenic ∆*spxB* mutants, grown to OD_600_ of 0.5, were analyzed by SDS-PAGE and western blot. A significant reduction in the amount of PLY released into the supernatants was observed in the ∆*spxB* mutant of each strain examined (Fig. 2 a-d). Differences in PLY release between wild-type and ∆*spxB* strains were not due to differences in colony forming units in the mid-log cultures as determined by plate counts (Additional file 2: Figure S2). Interestingly, addition of catalase to the medium only attenuated PLY release in high-releasing strains AW267 and WU2. Since catalase cannot cross the cell membrane and therefore can only neutralize extracellular H_2_O_2_, it is possible that the strains have different sensitivity to endogenous versus exogenous H_2_O_2_. Complementation of *spxB* restored the ability of strains to release significant concentrations of PLY (Fig. 2 e and f).

**Fig. 3:**
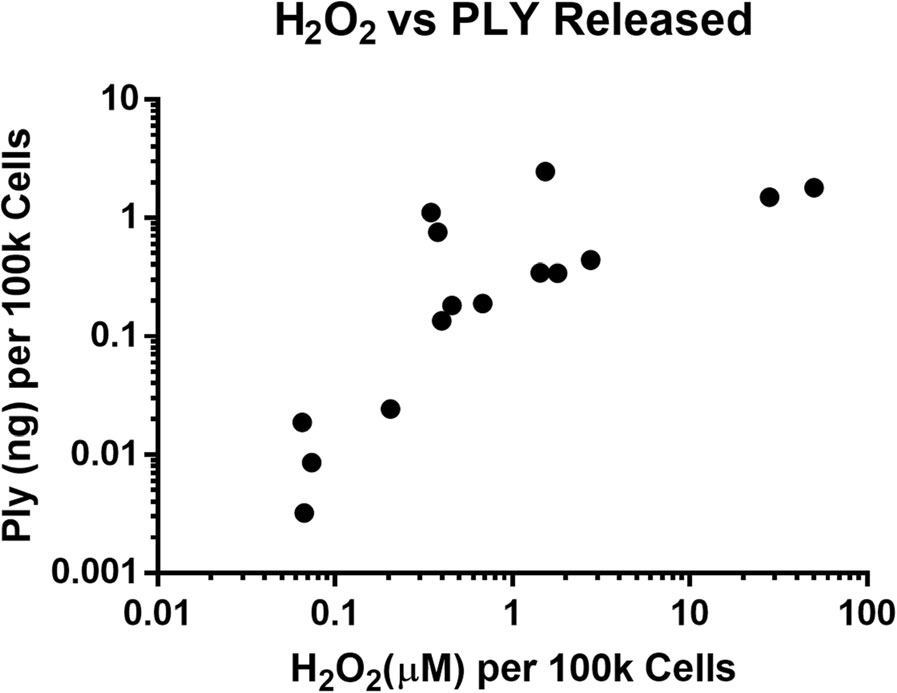
H_2_O_2_ production correlates with PLY release in clinical isolates. The amount of H_2_O_2_ and PLY in culture supernatants from 15 clinical isolates was measured by a colorimetric assay and dot blot, respectively. *Dot* intensities were quantiated using densitometry. PLY concentrations were interpolated using a standard line generated by serial dilution of recombinant PLY. Values for H_2_O_2_ and PLY were normalized to cell counts to account for differences in cell counts between strains and plotted versus each other. A linear regression was generated yielding a significant correlation (*p* < 0.05, *r* ^2^ = 0.3167)

To determine if PLY release correlated with H_2_O_2_ production in additional strains, a panel of 15 clinical isolates was analyzed. Each strain was grown to mid-log phase (OD_600_ of 0.5), serial dilutions of the culture were plated, and supernatants were used for and PLY and H_2_O_2_ quantitation. A dot blot assay was used to quantitate PLY as plotted against known concentrations of recombinant PLY. PLY and H_2_O_2_ concentrations were normalized to 100,000 cells and a linear scatter plot was generated (Fig. 3). A significant correlation was observed between H_2_O_2_ production and PLY released among the isolates examined (*r* ^2^ = 0.3167; *p* < 0.05). This suggests that H_2_O_2_ production impacts PLY release in clinically-relevant strains.

### Effects of SpxB on *ply* gene expression

**Fig. 4:**
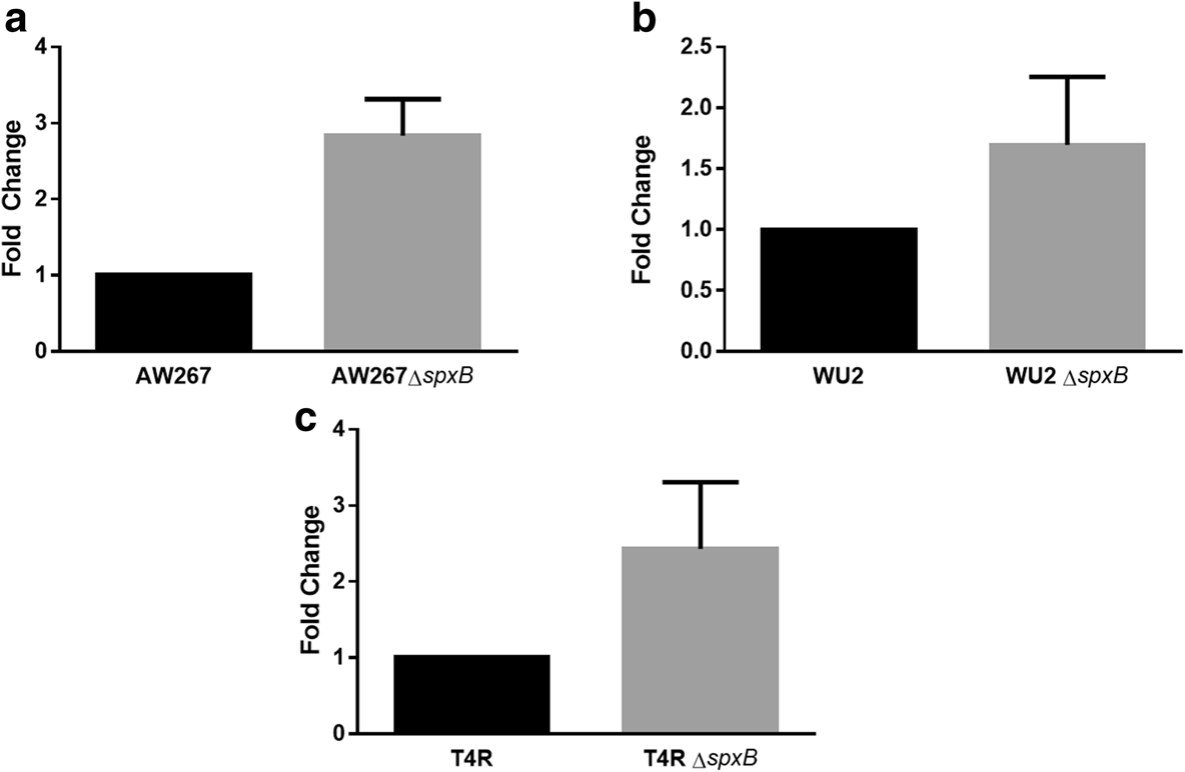
SpxB-dependent PLY release is not due to decreased transcription of the *ply* gene. Strains AW267 (**a**), T4R (**b**), WU2 (**c**), and their respective ∆*spxB* mutants were grown to mid-log phase and RNA was isolated and used for cDNA synthesis followed by qRT-PCR. GyrA was utilized as an internal control housekeeping gene. Fold-changes were quantitated by the 2^∆∆Ct^ method. Each figure represents three independent experiments ± SD

It is possible that expression of SpxB affects *ply* gene expression by metabolically altering the intracellular environment. To determine if deletion of *spxB* has a deleterious effect on *ply* gene transcription, leading to reduced amounts of PLY in the supernatant, qRT-PCR was performed on RNA isolated from strains grown to mid-log phase (Fig. 4). Surprisingly, the transcription of *ply* was found to be increased in all Δ*spxB* mutants analyzed (AW267∆*spxB*: 2.83-fold, WU2∆*spxB*: 1.96-fold, T4R∆*spxB*: 2.3-fold) which was in contrast to PLY release results shown in Fig. 2. Therefore, the reduced PLY release seen in Δ*spxB* mutants is not due to reduced transcription of the *ply* gene.

### Impact of exogenous H_2_O_2_ on PLY release

**Fig. 5:**
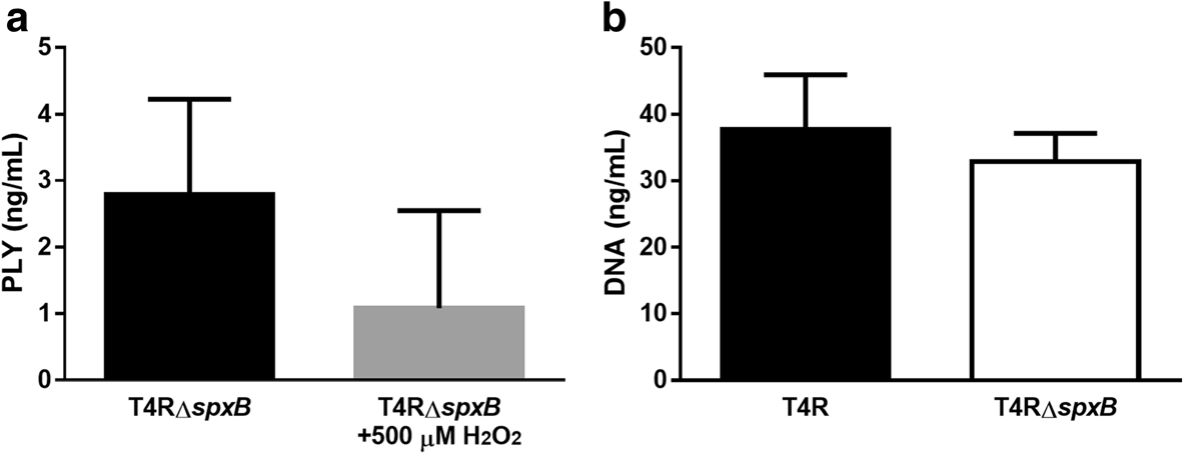
Exogenous H_2_O_2_ does not induce PLY release. **a** The ∆SpxB mutant of T4R was grown to an OD of 0.5. The bacterial cells treated with 500 μM H_2_O_2_ for 1 h and PLY concentrations were quantitated via dot blot. Each figure represents three independent experiments ± SD. **b** T4R and its ∆*spxB* mutant were grown to an OD_600_ of 0.5 and extracellular DNA was quantitated from sterile filtered supernatant by a Qbit fluorometer. Each figure represents three independent experiments ± SD

H_2_O_2_ is a by-product of SpxB activity and is known to have physiological effects upon pneumococcus. To determine the extent to which exogenous H_2_O_2_ can impact the release of PLY by inducing bactericidal lysis, T4R*∆spxB* was treated with either 0 μM or 500 μM H_2_O_2_ on ice for 1 h and the concentration of PLY in the supernatant was determined via dot blot. Interestingly, no significant difference was found between the H_2_O_2_-treated bacteria and those not receiving H_2_O_2_ (Fig. 5a). To ensure exposure to H_2_O_2_ did not impact bacterial survival, and therefore PLY production, bacterial colony forming units were enumerated prior to and after H_2_O_2_ exposure. Concentrations up to 500 μM did not impact bacterial survival (Additional file 3: Figure S3). Additionally, no significant difference in DNA release was seen with strains lacking *spxB* (Fig. 5b). These results, combined with the lack of catalase protection in the T4R strain (Fig. 2d), indicate that the contribution of SpxB to PLY release is not due to exogenous effects of H_2_O_2_ on bacterial viability and that H_2_O_2_ may exert its effects endogenously as it is being made.

### SpxB-dependent PLY release is not due to autolysis

**Fig. 6:**
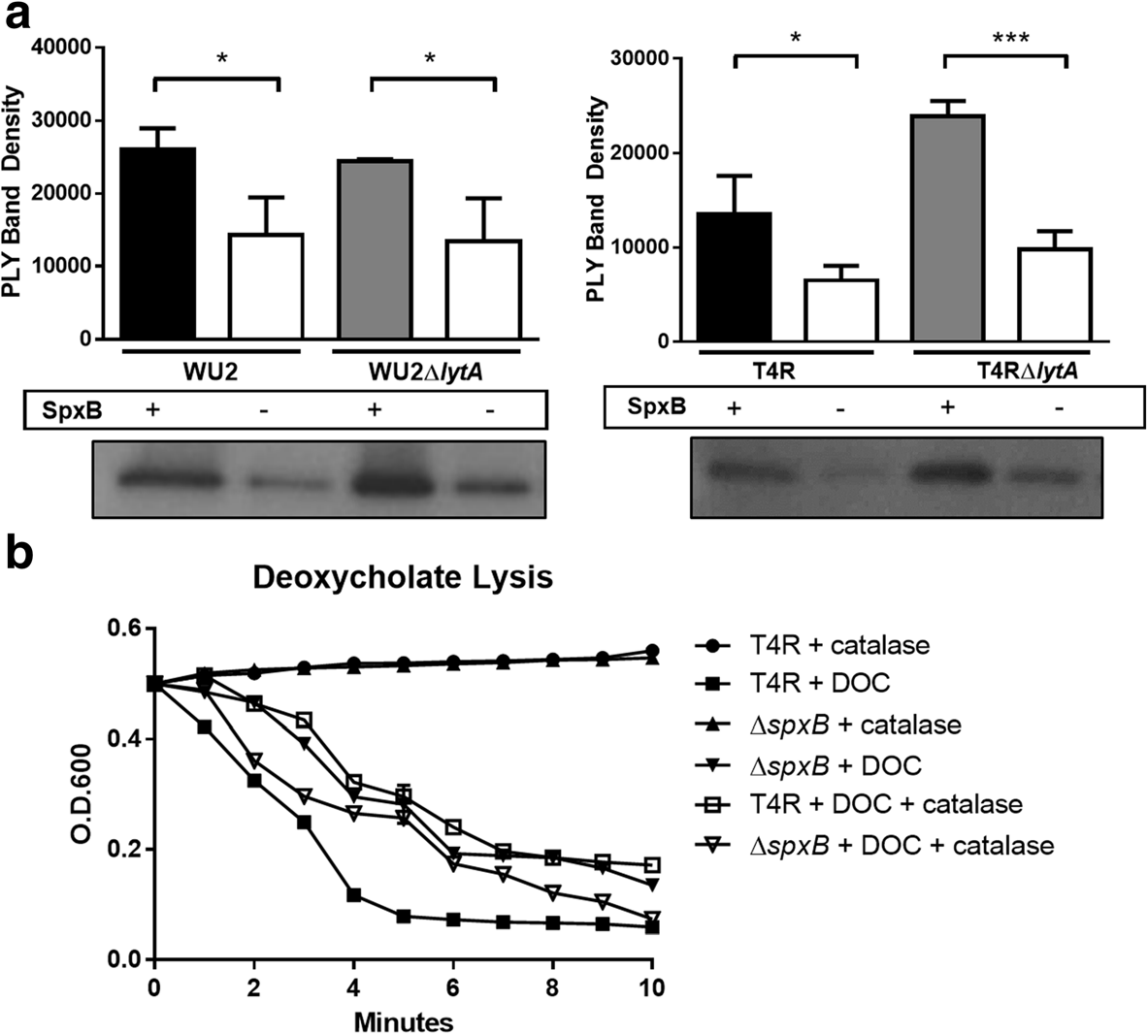
Effects of SpxB upon detergent-induced lysis. **a** WU2 and T4R along with their ∆*spxB*, ∆*lytA*, and ∆*spxB*/∆*lytA* mutants were grown to an OD of 0.5. Equal volumes of sterile supernatants were run on SDS-PAGE gels and probed for PLY via western blot. Each figure represents three independent experiments ± SD. (*p < 0.05). A representative western blot image for each strain is shown. **b** T4R and its ∆*spxB* mutant were grown to an OD of 0.5 prior to treatment with 0.05 % sodium deoxycholate (DOC). Spectrophotometric readings were taken every minute for 10 min to determine differences in the rate of induced autolysis

Another potential explanation for the difference in PLY release between the Δ*spxB* mutant and the parental strain is that loss of *spxB* leads to decreased autolysis even prior to stationary phase. Assays for autolysis were performed to determine if this was a contributing factor. Autolysis of *S. pneumoniae* involves the activity of the cell wall amidase LytA [34]. To determine whether LytA affected SpxB-dependent PLY release, western blots were performed on mid-log (OD_600_ of 0.5) supernatants from parental strains T4R and WU2 as well as isogenic mutants of both strains lacking either *lytA* or *spxB,* or both (Fig. 6a). Mutants lacking *spxB*, as expected, released less PLY, however loss of *lytA* had no significant effect on PLY release. These results indicate that the major autolysin, LytA, is not contributing to SpxB-induced PLY release at mid-log phase growth, but does not rule out its contribution at later times, such as during stationary phase.

It is possible that H_2_O_2_ or another by-product of SpxB activity could weaken the bacterial cell membrane, thereby facilitating PLY release. To determine if SpxB expression affected detergent-induced lysis, T4R and its isogenic ∆*spxB* mutant were grown to an OD_600_ of 0.5 and then treated with the ionic detergent sodium deoxycholate (DOC). As expected, addition of DOC to mid-log phase cultures initiated immediate lysis of both T4R and ∆*spxB* strains. However, wild type T4R lysed more rapidly and to a greater extent than T4R∆*spxB*. Supplementing the medium with catalase reduced lysis to levels comparable to T4R∆s*pxB*, indicating that H_2_O_2_ production may induce a condition that favors progression of lysis.

## Discussion

Pneumolysin is a major virulence factor of pneumococcus and its release into the extracellular space has been shown to vary greatly between strains [9, 35]. However, no single canonical mechanism of PLY release has been identified [8]. In this work, we have identified a novel link between expression of the metabolic enzyme pyruvate oxidase and release of PLY. Initially, we sought to determine the relative contribution of secreted H_2_O_2_ and PLY to host epithelial cytotoxicity. However, upon exposing A549 epithelial cells to pneumococcal supernatants, we found that supernatants from strains lacking SpxB showed significantly decreased cytotoxicity, due to reduced extracellular PLY release. Since H_2_O_2_ has been shown to be the primary cause of pneumococcal autolysis during stationary phase [27], we initially suspected the loss of viability by H_2_O_2_ produced by SpxB might cause the enhanced release of PLY in culture supernatant. However, there was no significant difference in colony forming units between parental and *spxB* mutants. Additionally, there was no significant difference in extracellular DNA concentrations between parental and ∆*spxB* mutants. Together, these results suggest that enhanced release of PLY in parental strains during mid-log phase growth was not due to a bactericidal effect of H_2_O_2_ produced by SpxB. While pneumococcal H_2_O_2_ is known to have cytotoxic effects on host cells [24, 26], our results indicate that the loss of host cell integrity is primarily linked to PLY released into the supernatant, not to H_2_O_2_. However, it is possible that SpxB-dependent H_2_O_2_ induces stress within epithelial cells that may lead to alternative, non-cytolytic death processes. For instance, it has been shown that SpxB-induced H_2_O_2_ triggers genotoxicity and conserved stress responses within the epithelium [24, 36].

While H_2_O_2_-induced bactericidal effects do not appear to be the mechanism for PLY release, our findings do indicate that release may be linked to the production of H_2_O_2_, as indicated by the correlation between PLY release and H_2_O_2_ production in our clinical isolate panel. However, SpxB enzymatic activity produces other byproducts, including acetate and acetylphosphate that could potentially contribute to this mechanism of release [20]. Acetyl-phopshate contributes significantly to the ATP pool of pneumococcus [19]. We are currently investigating whether PLY release is energy-dependent. Furthermore, the addition of catalase to the culture medium abrogated PLY release from some strains (WU2), while failing to prevent release from others (TIGR4). These results suggest that strain-specific differences in metabolism might impact the release of PLY. We are also currently investigating how the capsule genes possessed by different serotypes may alter their metabolic processes and thereby SpxB activity and subsequent PLY release. However, if H_2_O_2_ serves to release PLY, it appears that it may act from within the bacterial cell, as supplementation of exogenous H_2_O_2_, surprisingly, did not enhance PLY release, at least in the T4R strain. Also, while both *spxB* and *ply* are known to be differentially expressed in different biological niches during infection [37], *ply* was not found to be expressed greater in parental strains as compared to ∆*spxB* strains.

Our results indicate that the PLY release mechanism appears to be autolysis-independent, as mutants lacking the major autolytic cell wall amidase LytA were equally able to release PLY as parental strains. Furthermore, double mutants lacking both SpxB and LytA released equal PLY to strains lacking only SpxB. However, it is possible that additional autolytic factors including the murein hydrolase LytB, competence induced bacteriocin (CibAB), or choline binding protein D (CbpD) could play a role in *spxB*-dependent PLY release [38–40]. Results from exposure of the parental strain T4R to deoxycholate indicate that *spxB* expression may predispose the bacterial cells to autolysis. This indicates that H_2_O_2_ or some other SpxB-induced byproduct could weaken the bacterial cell membrane allowing for release of PLY from the intracellular space, possibly in the absence of cell death. However, loss of cell membrane integrity would likely free intracellular stores of LytA which could trigger autolytic effects not seen in our studies [41, 42]. An intriguing possibility is that PLY possesses characteristics that allow it to preferentially traverse the SpxB-perturbed membrane. We are currently investigating whether other cryptically secreted proteins may escape the cytosol via the SpxB-dependent mechanism used by PLY.

We have yet to determine is whether or not H_2_O_2_ produced by SpxB impacts pneumococcal activators or repressors, thereby indirectly affecting expression of genes that could impact PLY release. In *Staphylococcus aureus,* exposure to exogenous H_2_O_2_ enhanced expression of multiple oxidative stress response genes controlled by the ferric uptake regulator (Fur) homolog PerR [43]. While pneumococcus doesn’t possess PerR, the iron uptake regulator RitR regulates expression of a number of genes which are impacted by both iron availability and oxidative stress [44]. The MerR regulators of pneumococcus are another group of regulatory proteins that can be impacted by oxidative stress [45].

Since SpxB is an important metabolic enzyme for the organism during aerobic growth, our findings could link cytotoxic potential of strains to metabolic shifts that occur at different niches throughout the host during colonization or progression to invasive disease. This mechanism could constitute another reason for the attenuated virulence seen with *spxB*-negative mutants [21]. However, it was recently demonstrated that increased PLY release does not necessarily translate to enhanced virulence [16]. This underlines SpxB as an important virulence regulator, as our data indicate that SpxB expression contributes significantly to the release of PLY at early phases of growth, along with previous data indicating that it allows the organism to outcompete other common inhabitants of the nasopharynx [22]. These findings could represent a novel method for protein secretion that extends beyond pneumococcus to other bacterial species.

## Conclusions

Expression of SpxB was shown to contribute to PLY release in various *S. pneumoniae* strains. While a panel of clinical isolates demonstrated a correlation between H_2_O_2_ production and PLY release, catalase was able to prevent PLY release in a strain-dependent manner. This indicates endogenous/exogenous H_2_O_2_ may contribute in a strain-dependent fashion to SpxB-dependent PLY release. PLY release due to SpxB was not dependent upon cellular turnover based on plate counts and DNA release. Though SpxB-dependent PLY release was not dependent on the activity of the cell wall amidase LytA, deoxycholate-induced autolysis was greater in strains expressing SpxB, indicating possible weakening of the cell membrane when SpxB is expressed. These results identify a novel route of PLY release that may extend to secretion of other cytoplasmic proteins that lack signal sequences.

## Additional files

Additional file 1: Figure S1. Deletion of *spxB* greatly reduces production of hydrogen peroxide. The amount of hydrogen peroxide in culture supernatants from wild type and mutant strains lacking SpxB (A. AW267; B. WU2; C. T4, D. T4R) was measured via a colorimetric peroxide assay. Asterisks indicate statistical significance (**** *p* < 0.00005, ***p* < 0.005) and ND indicates a peroxide concentration below the detectable limits of the assay. (TIF 534 kb)
Additional file 2: Figure S2. Bacterial colony counts. Serial dilution plate counts were made at OD 0.5 of the indicated strains to determine if there was a difference in the bacterial counts when SpxB is removed, or when catalase is added. Each figure represents three independent experiments ± SD. (TIF 540 kb)
Additional file 3: Figure S3. Bacterial colony counts. Serial dilution plate counts were made from cultures grown to OD_600_ 0.5 prior to and 1 h post-exposure to various concentrations of H_2_O_2_. Results are shown as the average of 3 independent experiments ± SD. (TIF 481 kb)

## Abbreviations

H_2_O_2_: Hydrogen peroxide
LytA: Autolysin
PLY: Pneumolysin
SpxB: Pyruvate oxidase
