# Supplementary figures and images for: Pyruvate oxidase of Streptococcus pneumoniae contributes to pneumolysin release
Source: BMC Microbiol. 2016 Nov 9;16:271. doi: 10.1186/s12866-016-0881-6 (PMC5103497; doi:10.1186/s12866-016-0881-6)

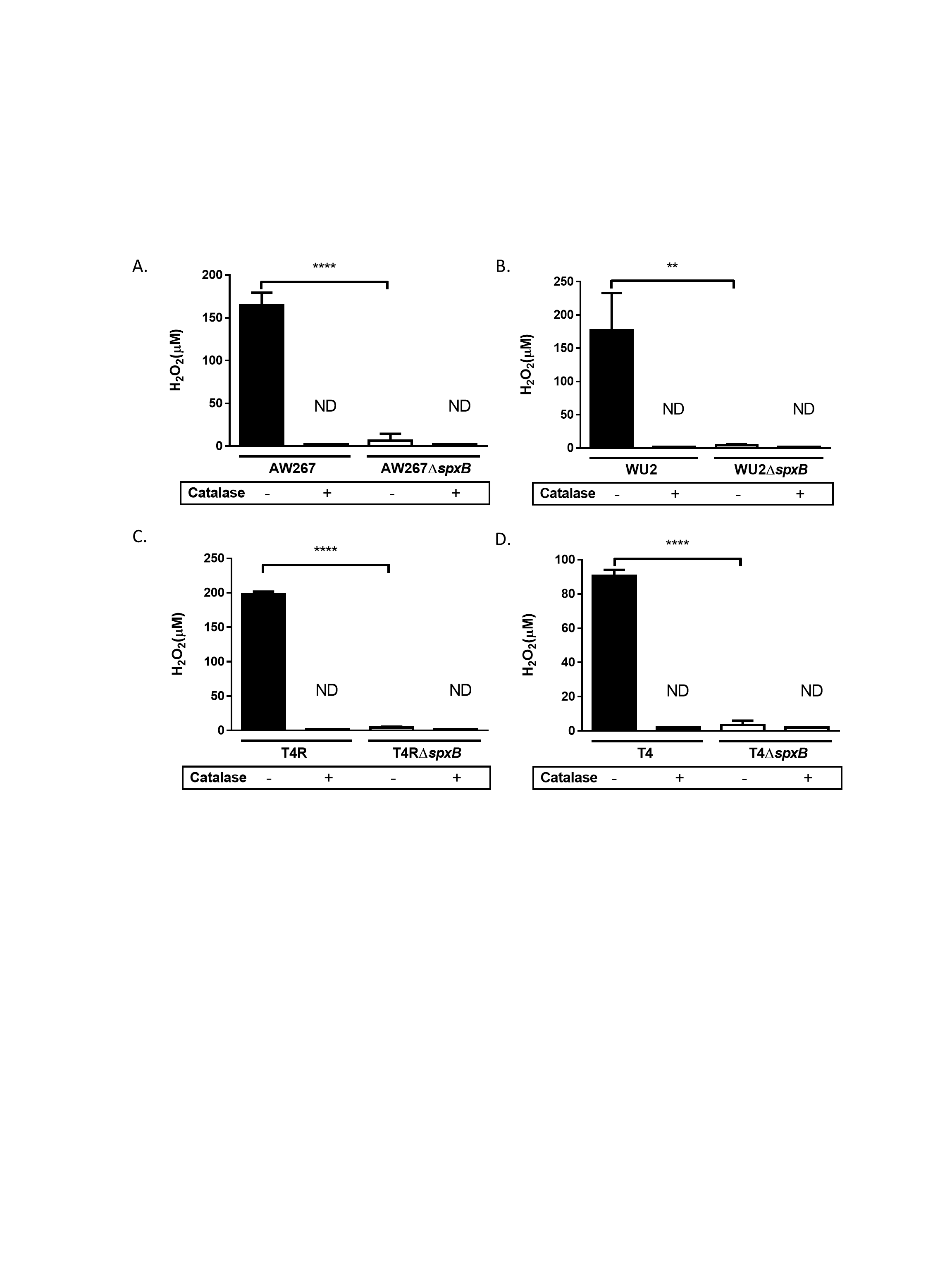

Supplement: Additional file 1: Figure S1. — Deletion of spxB greatly reduces production of hydrogen peroxide. The amount of hydrogen peroxide in culture supernatants from wild type and mutant strains lacking SpxB (A. AW267; B. WU2; C. T4, D. T4R) was measured via a colorimetric peroxide assay. Asterisks indicate statistical significance (**** p < 0.00005, **p < 0.005) and ND indicates a peroxide concentration below the detectable limits of the assay. (TIF 534 kb) [file 12866_2016_881_MOESM1_ESM.tif]

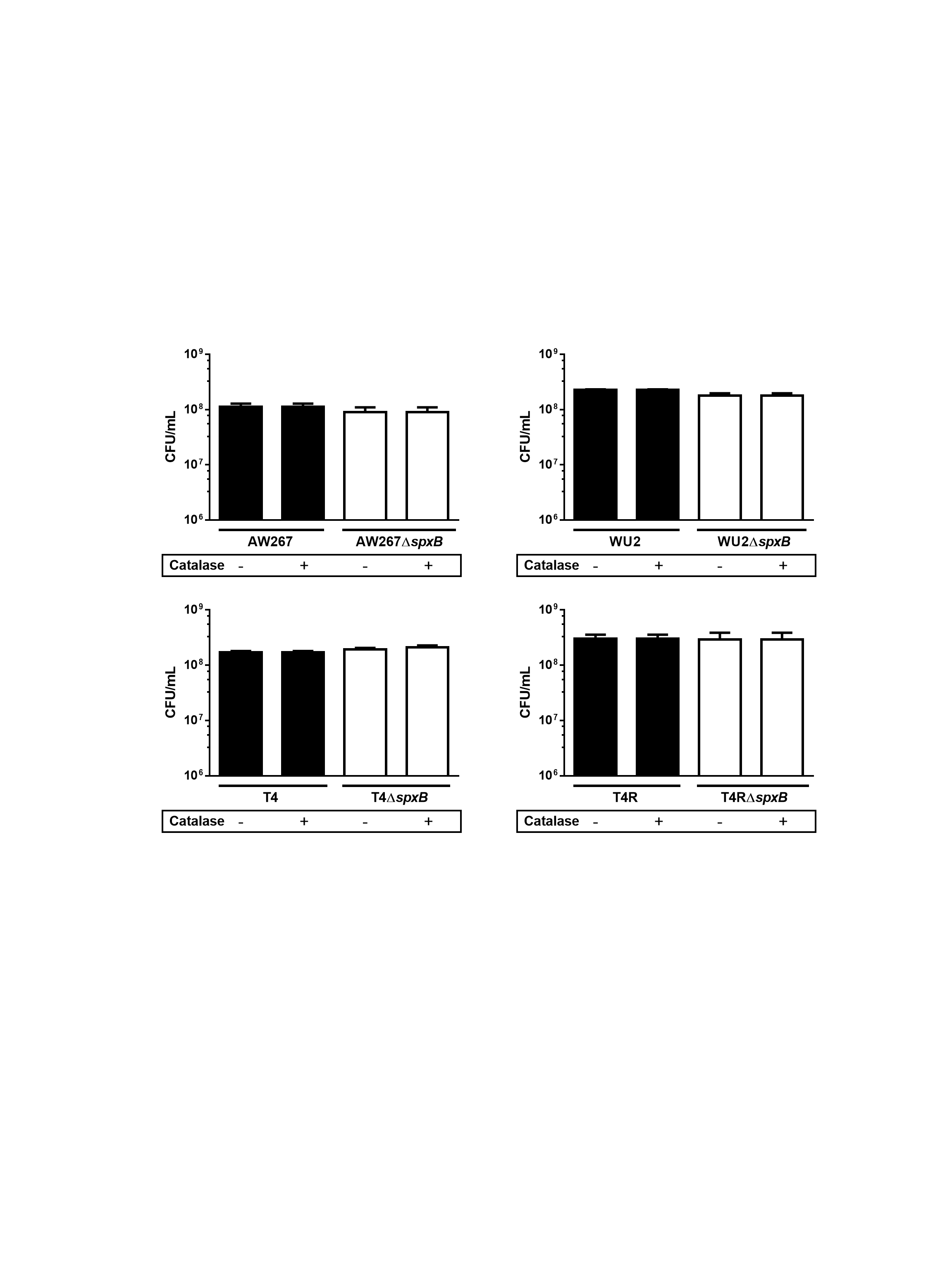

Supplement: Additional file 2: Figure S2. — Bacterial colony counts. Serial dilution plate counts were made at OD 0.5 of the indicated strains to determine if there was a difference in the bacterial counts when SpxB is removed, or when catalase is added. Each figure represents three independent experiments ± SD. (TIF 540 kb) [file 12866_2016_881_MOESM2_ESM.tif]

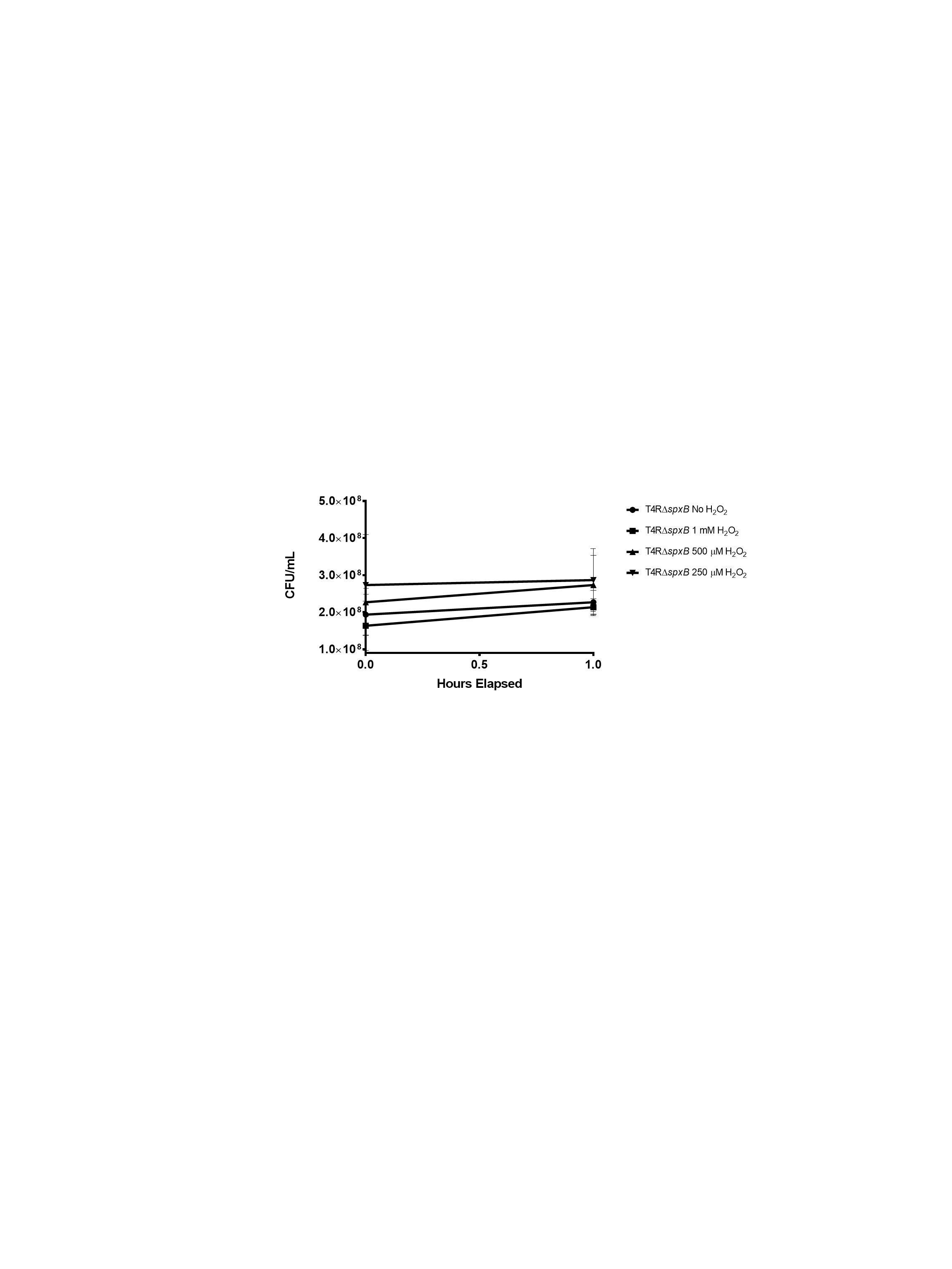

Supplement: Additional file 3: Figure S3. — Bacterial colony counts. Serial dilution plate counts were made from cultures grown to OD600 0.5 prior to and 1 h post-exposure to various concentrations of H2O2. Results are shown as the average of 3 independent experiments ± SD. (TIF 481 kb) [file 12866_2016_881_MOESM3_ESM.tif]
